# Selective Sweeps Uncovering the Genetic Basis of Horn and Adaptability Traits on Fine-Wool Sheep in China

**DOI:** 10.3389/fgene.2021.604235

**Published:** 2021-02-23

**Authors:** Tingting Guo, Hongchang Zhao, Chao Yuan, Shuhong Huang, Shiwei Zhou, Zengkui Lu, Chun’e Niu, Jianbin Liu, Shaohua Zhu, Yaojing Yue, Yuxin Yang, Xiaolong Wang, Yulin Chen, Bohui Yang

**Affiliations:** ^1^Animal Science Department, Lanzhou Institute of Husbandry and Pharmaceutical Sciences, Chinese Academy of Agricultural Sciences, Lanzhou, China; ^2^Key Laboratory of Animal Genetics, Breeding and Reproduction of Shaanxi Province, College of Animal Science and Technology, Northwest A & F University, Xianyang, China; ^3^Engineering Research Center of Sheep and Goat Breeding, Chinese Academy of Agricultural Sciences, Lanzhou, China

**Keywords:** fine-wool sheep, whole-genome resequencing, selective sweeps, genetic basis, horn, adaption

## Abstract

Long-term natural and artificial selection leads to change in certain regions of the genome, resulting in selection signatures that can reveal genes associated with selected traits, such as horns (i.e., polled/horned), high-quality wool traits, and high-altitude hypoxia adaptability. These are complex traits determined by multiple genes, regulatory pathways, and environmental factors. A list of genes with considerable effects on horn and adaptability traits has not been found, although multiple quantitative trait loci (QTL) have been identified. Selection signatures could be identified using genetic differentiation (F_*ST*_), polymorphism levels θπ, and Tajima’s D. This study aimed to identify selection signatures in fine-wool sheep and to investigate the genes annotated in these regions, as well as the biological pathways involved in horn and adaptability traits. For this purpose, the whole-genome sequence of 120 individuals from four breeds, which come from different elevations and habitats in China, was used to analyze selection signatures for horn and adaptability traits. Annotation of the consensus regions of F_*ST*_ and θπ ratios revealed a list of identified genes associated with polled/horned and high-altitude hypoxia adaptability traits, such as *RXPF2*, EE*RFC4*, *MSH6*, *PP1R12A*, *THBS1*, *ATP1B2*, *RYR2*, and *PLA2G2E*. Gene ontology (GO) and Kyoto Encyclopedia of Genes and Genomes (KEGG) analysis identified genes related primarily to mismatch repair, metabolism, vascular smooth muscle contraction, and cardiac muscle contraction. This is the first study to demonstrate that selection signatures play an important role in the polled/horned and high-altitude hypoxia adaptability traits of fine-wool sheep breeds that have undergone high-intensity selection and adapted to different ecological environments in China. Changes observed in the genome of fine-wool sheep may have acted on genomic regions that affect performance traits and provide a reference for genome design and breeding.

## Introduction

Sheep (*Ovis aries*) have played an important role in human society as one of the first animals ever domesticated, and have spread almost globally via human migrations. Sheep were initially reared by humans mainly for mutton to meet their survival needs, and were the main source of meat for people living in remote and nomadic areas. With the improvement of people’s living standards and the continuous enrichment of material needs, fine-wool sheep emerged later as a specialization for secondary products, such as wool. Fine-wool sheep are renowned for their ability to produce high-quality wool, a natural fiber that is an important agricultural commodity used in clothing and textiles. The wool traits of fine-wool sheep have always been the focus of artificial selection in breeding. However, their high-altitude adaptability has also been subject to natural selection over many years, because of the extreme differences between the ecological environments in which they live. For example, some fine-wool sheep live in desert grassland plateaus, and others in high-altitude pastoral areas, while yet others live in low-altitude agricultural areas or desert regions. Thus, it is important to understand the genetic basis of well-adapted local livestock breeds in extreme environments to develop appropriate breeding programs under scenarios of future climate change (Li et al., 2014). Sheep have adapted to a wide range of agroecological conditions and are sensitive to climate change (Lv et al., 2015). Thus, fine-wool sheep provide an excellent model to gain novel insights into genetic mechanisms underlying the rapid adaptations of livestock to extreme environments within a short period of time.

In recent years, the mechanisms of organisms’ adaptation to the low oxygen levels in high-altitude areas are of great interest, and whole-genome sequencing studies have been performed on a wide range of organisms that live in harsh or extreme environments to characterize adaptive genetic variation (Simonson et al., 2010; Mackelprang et al., 2011; Scheinfeldt et al., 2012; Cho et al., 2013; Ge et al., 2013; Ma et al., 2013; Qu et al., 2013; Liu et al., 2014). Studies conducted on livestock are limited, especially in goats and sheep. Goats and sheep are versatile domestic species that have been integrated into diverse environments and production systems (Kim et al., 2016). However, to our knowledge, no study has characterized the rapid genetic adaptations of fine-wool sheep to various extreme environments based on whole-genome sequences.

Horns are crucial for the survival of wild sheep because strong horns are advantageous in competition for mating, and horns are an essential defense against predators. The RXFP2 gene has undergone rapid evolution. In most domestic sheep, horns become vestigial because traits that ensure fitness in the wild often become useless under conditions of domestication. Studies have shown that the *RXFP2* haplotype was significantly associated with horn size and shape. *RXFP2* is a well-known genetic determinant of horn phenotype in sheep. This locus is correlated with quantitative and discrete traits of horns in wild and feral populations (Johnston et al., 2011, 2013; Kardos et al., 2015). For domestic sheep, single-nucleotide polymorphisms (SNPs) within or around *RXFP2* are predictive for polled/horned traits (Dominik et al., 2012; Wiedemar and Drögemüller, 2015). Although the contributions of *RXFP2* to horn phenotypes have been extensively studied (Johnston et al., 2011; Kijas et al., 2012; Kardos et al., 2015), little is known about the mechanism that accounts for fine-wool sheep horns. These sheep have undergone rapid evolution across the RXFP2 gene to acquire very strong and weapons-grade horns, as a consequence of sexual selection and reduced human intervention. Whether other genes have been selected to control polled/horned traits during the breeding of intensively artificially bred fine-wool sheep has not been studied.

In the present study, we sequenced the genomes of 120 fine-wool sheep representing four fine-wool sheep breeds (alpine merino sheep, Chinese merino sheep, Aohan fine-wool sheep, and Qinghai fine-wool sheep) of different genetic and geographic origins, from habitats in different high-altitude environments in China (Supplementary Table S1). The whole-genome sequence data from these fine-wool sheep were subsequently analyzed, the genomes of sheep from different high-altitude environments were aligned with those from different ecological environment ecotope, and the genetic variation in genomic regions that show evidence of selection was explored further based on selective sweeps. We aimed to identify the candidate genes, functional gene ontology (GO) categories, and signaling pathways responsible for the presence or absence of horns (i.e., horned/polled) and the rapid adaptations of sheep to the high-altitude plateau, to elucidate the population genetics and adaptation to high altitude, presence/absence of horns, and other traits.

## Materials and Methods

### Ethics Statement

All experimental procedures were performed in accordance with the Regulations for the Administration of Affairs Concerning Experimental Animals approved by the State Council of People’s Republic of China. The study was approved by the Institutional Animal Care and Use Committee of the Lanzhou Institute of Husbandry and Pharmaceutical Sciences at the Chinese Academy of Agricultural Sciences.

### Sample Collection and Whole-Genome Sequencing

Blood samples were collected from 30 fine-wool sheep of each breed (2-year-old rams from different families) that include four representative breeds [alpine merino sheep (AMS), Chinese merino sheep (CMS), Aohan fine-wool sheep (AHS), and Qinghai fine-wool sheep (QHS)] adapted to various altitudes local environments. A total of 120 blood samples are collected from the origin preserving farm or the core flock. The environments included here represent the typical environments that fine-wool sheep inhabit. For a description of the different breeds or populations, see Supplementary Table S1, such breeds, numbers sampled, origin, sex, horned or polled, and altitude.

All the sampled blood was preserved in blood-collecting vessels (Jiangsu Yuli Medical Instrument Co., Ltd, China) and stored at −20°C until DNA extraction. Genomic DNA was extracted from the blood using a TIANamp Genomic DNA Kit according to the manufacturer’s instructions. All the DNA samples were sequenced using the Illumina HiSeq X10 sequencer in paired-end 150 bp mode at Novogene (Beijing, China); 32 samples were sequenced at a depth of 30 (while the remaining 88 were sequenced at a depth of 5).

### SNP Calling and Annotation

High-quality reads were aligned against the reference sheep genome assembly Oar_v4.0 (GCF_000298735.2) using Burrows-Wheeler Aligner (BWA) software (v0.7.11) (Li and Durbin, 2009), with the following alignment parameters: mem -t 4 -k 32 -M. The SAM tools (v0.1.19) program was used to identify single-nucleotide polymorphisms (SNPs) (Li et al., 2009) and to exclude SNP-calling errors caused by incorrect mapping. The eligible SNPs were then filtered according to the following criteria: (i) coverage depth >3, (ii) missing ratio of samples within population <20, and (iii) minor allele frequency (MAF) >5%. SNPs obeyed after the above standards proceed to statistics analysis. The SNPs were annotated using ANNOVAR software with the default parameter settings (Wang et al., 2010).

### Population Genetics Analysis

#### Principle Component Analysis

Principle component analysis (PCA) was performed based on all SNPs using EIGENSOFT software (v4.2) (Patterson et al., 2006). This package applies PCA to genetic data to analyze the population structure. The samples were clearly separated into four groups by the first principal component. The figures were then plotted using the first and second principal components with using the built-in R functions prcomp () and princomp ().

#### Phylogenetic Trees

TreeBeST software (v1.9.2) was used to construct the phylogenetic tree (bootstrap values were obtained after 1000 calculations) via the neighbor-joining method (Saitou and Nei, 1987).

#### Structural Analysis

The ancestry of each individual was estimated using the genome-wide unlinked SNP data set and the model-based assignment software Admixture (v1.3) (Alexander and Lange, 2011) to quantify genome-wide admixture between the sheep breeds. Admixture was run for each possible group number (K (( 2 to 6) with 200 bootstrap replicates to estimate the parameter standard errors used to determine the optimal group number (K).

#### Population Linkage disequilibrium

Based on the non-inbred individuals [identity-by-state (IBS) < 0.9], we calculated the linkage disequilibrium (LD) decay for different breeds via the squared correlation coefficient (*r*^2^) between any two loci using Haploview (v4.2) (Barrett et al., 2005). Average *r*^2^ was calculated for pairwise markers in a 20-kb window and averaged across the whole genome.

### Detection of Selective Signatures

In order to detect the positive selection effect, we compared the difference of population mutation rate θ*w* (reflecting the base mutation rate of the population’s genome) and (((reflecting the base diversity of the population’s genome) in every breed. To identify the signals of selection, the genome-wide fixation index (FST),Tajima’s D and ((ratios (((low/((high and ((horned/((polled) were calculated using a sliding window approach (100-kb window sliding in 50-kb steps) (Korneliussen et al., 2013; Fumagalli et al., 2014). To detect the genomic loci that are associated with horn and adaptation, we also calculated the pairwise F_*ST*_ and ((ratios (((low/((high and ((horned/((polled). To reduce the number of false positives, only the outlier windows showing extremely high FST and ((ratio (corresponding to the 5% left and right end of the tail) were identified as selection signals. Also, we calculated the heterozygosity (Hp) of SNP sites within the window and Z-transformed Hp and FST. According to the value of each window, select windows ZHp ≤ 0 and ZF_*ST*_ ≥ 0 to draw the distribution map on the reference genome.

### Functional Annotation

According to genome annotation, a gene was assumed to be under positive selection if it overlapped with a selection signal. To obtain an in-depth view of the biological significance of the candidate selection signals in each breed, we carried out Gene Ontology (GO) and Kyoto Encyclopedia of Genes and Genomes (KEGG) functional enrichment analysis. Significant enrichment analysis of pathways can determine the most important biochemical metabolic pathways and signal transduction pathways involved in candidate genes.

### Candidate Genes Analysis

Individual haplotype blocks and haplotype frequency were detected using the standard expectation maximization (EM) (Barrett et al., 2005) algorithm. Reference thresholds and partitioning criteria for SNPs within closely linked haplotypes were established following the recommendations of the user manual for PLINK software (Purcell et al., 2007). The recessive multi-alleles were used as a random effect in the association study model. In addition, genotypic, allelic frequencies and genetic parameters, including the polymorphism information content (*PIC*), effective allele number (*Ne*), and expected heterozygosity (*He*) (Watterson, 1975), as well as Hardy–Weinberg equilibrium (*HWE*) testing *p*-value, were directly calculated (Zhao et al., 2013). Tajima’s D and fixation statistics (Fst) values were calculated for detecting selection signature of candidate genes.

## Results

### Genome Resequencing and Genetic Variation

We generated whole-genome sequences of 120 fine-wool sheep from four independent populations at an average depth of 5×, a total of 2,206 GB raw data were obtained, and after filtering, 2,203 GB of clean data were retained (Supplementary Tables S2, S3). We identified 4,824,792 SNPs among these four breeds (Table 1). Additionally, we generated whole-genome sequences of 32 fine-wool sheep from four independent populations at a mean depth of 30×, totaling 3,426 GB raw data and after filtering 3,422 GB clean data; 18,898,476 SNPs were identified (Table 2).

**TABLE 1:** Characteristics and numbers of identified SNPs for 120 individuals of four breeds fine-wool sheep (sequencing depth 5×).

Category		Number of SNPs
Upstream		36,395
Exonic	Stop gain	261
	Stop loss	30
	Synonymous	39,547
	Non-synonymous	22,869
Intronic		1,859,102
Splicing		172
Downstream		35,884
Upstream/downstream		1,044
Intergenic		2,733,827
Transformation		3,567,937
Transversion		1,256,855
Transformation/transversion		2.838
Total		4,824,792

**TABLE 2:** Characteristics and numbers of identified SNPs for 32 individuals of four breeds fine-wool sheep (sequencing depth 30×).

Category		Number of SNPs
Upstream		103,550
Exonic	Stop gain	423
	Stop loss	61
	Synonymous	69,425
	Non-synonymous	40,143
Intronic		5,950,716
Splicing		238
Downstream		103,590
Upstream/downstream		1,298
Intergenic		12,617,424
Transformation		13,598,912
Transversion		5,299,564
Transformation/transversion		2.566
Total		18,898,476

### Population Genetics Structure

**FIGURE 1:**
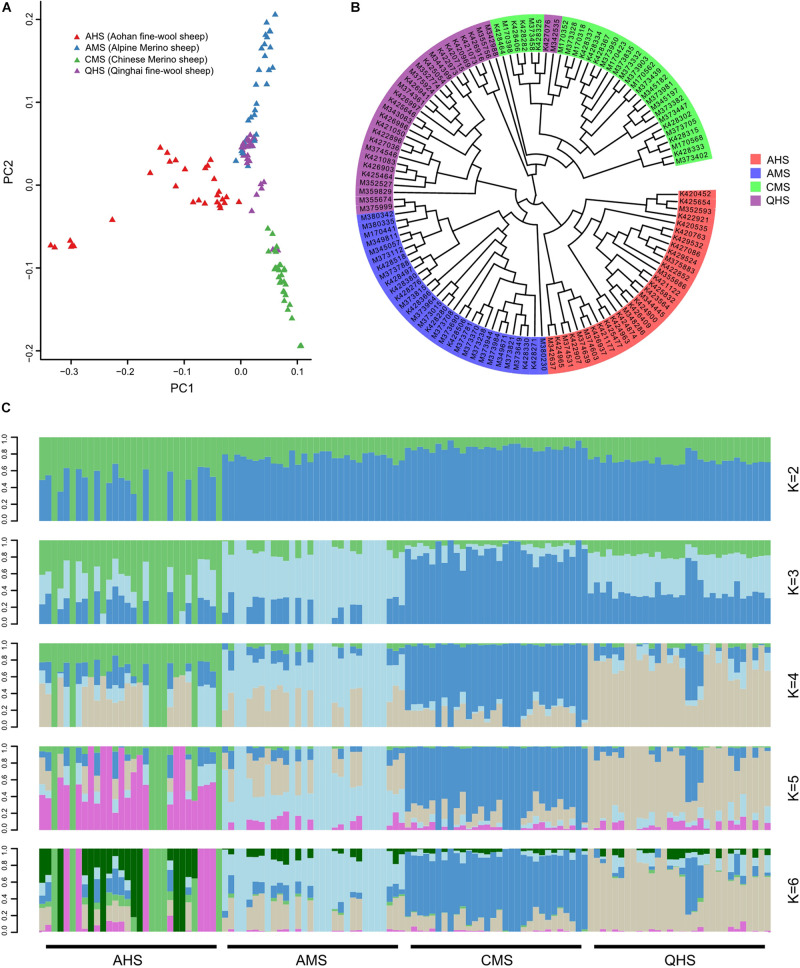
Population genetics structure. **(A)** Principal component analysis of identified SNPs. **(B)** Neighbor-joining (NJ) tree. **(C)** Genome-wide admixture analysis inferred from SNPs.

To examine genetic relationships among fine-wool sheep in China, the PCA (Figure 1A), neighbor-joining (NJ) phylogeny (Figure 1B), and Admixture (*K* = 6) analyses based on the the whole-genome sequencing data. SNPs seats yielded indicate the major genetic division among the fine-wool sheep from four large geographic regions of differing altitudes (Figure 1C). PCA explained the proportion of total variance by each principal component: 2.08, 1.94, and 1.85% for principal component 1 (PC1), PC2, and PC3, respectively. PCA clearly showed that Aohan fine-wool sheep (AHS) was clearly partitioned from alpine merino sheep (AMS) and Chinese merino sheep (CMS). However, Qinghai fine-wool sheep (QHS) was not completely separated from AMS and CMS, which may be related to the joint breeding of QHS with AMS and CMS (Di et al., 2011, Di et al., 2014; Li et al., 2016; Zhao et al., 2019; Wei et al., 2020). To resolve their phylogenetic relationships, we constructed a NJ tree among the 120 individuals based on the pairwise genetic distances (Figure 1B). Aohan fine-wool sheep (AHS) were found in a unique branch. The other main lineage of the NJ tree included all AMS and most QHS. In addition, all CMS and several QHS were found in one branch. The results were similar to PCA, showing that AMS, QHS, and CMS are closely related, and all three breeds have Australian merino ancestry. Additionally, QHS has CMS ancestry. The Admixture analysis also supported these findings. Considering possible admixture among the breeds, we further performed population structure analysis using Admixture software (Tang et al., 2005), which estimates individual ancestry and admixture proportions assuming K ancestral populations (Figure 1C). To confirm our observation of the degree of divergence, the Admixture software was applied to estimate the proportion of common ancestry among the four breeds. A model-based unsupervised hierarchical clustering of the individuals was analyzed by considering different *K* values (2–6) of predefined clusters. The results of Bayesian clustering for *K* = 2 indicated that there was a clear division between the breeds living at low altitude (AHS) and at middle/high altitude (CMS, AMS, and QHS). This is also consistent with the PCA and NJ tree. Furthermore, when the *K* value became large, some breeds were independent. At *K* = 3, the breeds living at middle altitude (CMS) tended to be separated from those living at high altitude (AMS and QHS). At *K* = 4, AMS and QHS were separated, although there was a slight ingression of QHS in the CMS breed. We observed a clear signature of genetic admixture among four sheep breeds, as well as genetic introgression from eastern to northern stock in Chinese fine-wool sheep breeds.

### Population Genetics Analysis

**FIGURE 2:**
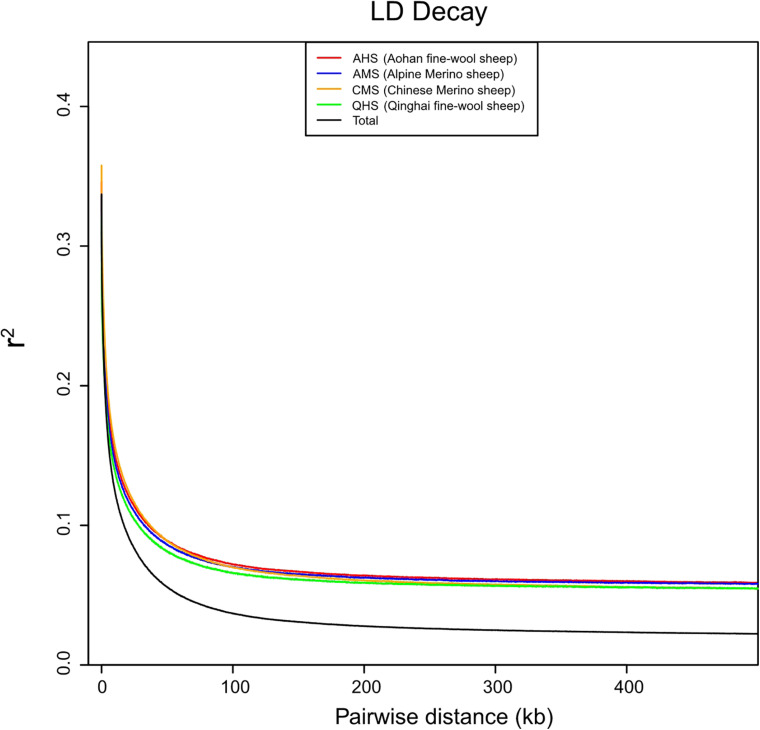
Population selective analysis of linkage disequilibrium (LD) decay. The LD decay rate was measured as the distance at which the average squared correlations of allele frequencies (*r*^2^) dropped to half its maximum value.

To further study the degree of selection of different fine-wool breeds, we separated the four groups and calculated the LD values of each group and all groups together. The LD analysis showed that QHS had the lowest LD decay rate of all the breeds (Figure 2). Generally, the lower the degree of domestication and the stronger the selection of the population *were*, the *slowest* LD decay rate *was*. These results indicate that QHS has been artificially bred at a lower selection intensity than other breeds, leading to a higher genetic diversity. The nucleotide variability and polymorphism (θ*w* and θπ) analysis has been shown to be an effective method for detecting population genetic diversity, especially in the mining of the course of domestication and improvement closely related to the habitat. Nucleotide variability and polymorphism (θ*w* and θπ) in each population were analyzed by sequence diversity statistics (Nei and Li, 1979). In this study, we found that θπ>θ*w* in CMS indicates that CMS has higher nucleotide variability and polymorphism (θ*w* and θπ) than other breeds (Table 3), which means that the genetic diversity is higher than others.

**TABLE 3:** The nucleotide variability and polymorphism (θ*w* and θπ) in each population.

Sample	θπ	θ*w*
AHS	3.838E-4	3.925E-4
AMS	3.524E-4	3.930E-4
CMS	4.890E-4	3.954E-4
QHS	3.288E-4	3.938E-4

**FIGURE 3:**
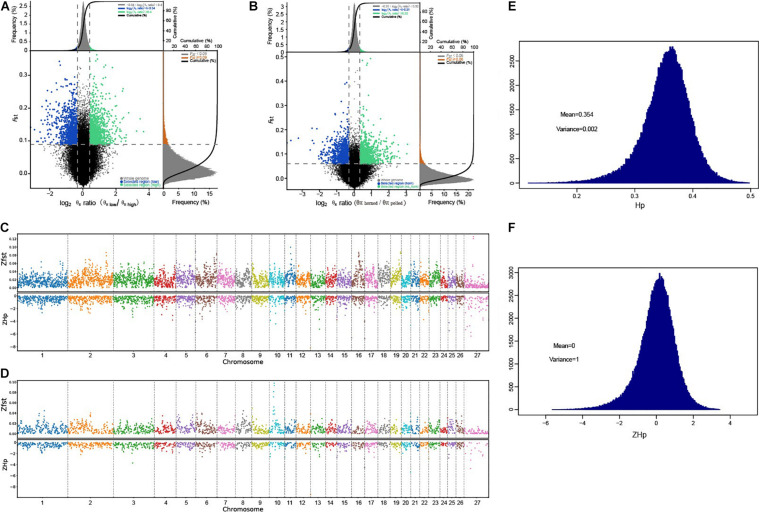
Selection sweep analysis. **(A)** FST and θπ ratio selective elimination analysis chart for adaptive traits. **(B)** FST and θπ ratio selective elimination analysis chart for horn traits. The abscissa is the θπ ratio value, and the ordinate is the FST value, corresponding to the frequency distribution diagram above and the frequency distribution diagram on the right, respectively, and the dot diagram in the middle represents the corresponding FST and θπ ratio values in different windows. The blue and green areas are the top 5% areas selected by θπ and FST. **(C)** Distribution of ZFST and ZHp on the chromosome for adaptive traits. **(D)** Distribution of ZFST and zHp on the chromosome for horn traits. The larger the ZFST value (upper half), the more obvious the species differentiation and the higher the degree of selection. The smaller the zHp value (lower half), the smaller the heterozygosity of the species in this window, indicating a higher degree of selection. **(E)** Distribution of Hp scores. The abscissa indicates the heterozygosity rate, and the ordinate indicates the heterozygosity rate distribution; μ, mean, σ and standard deviation. **(F)** Standardized normal distribution of Z-transformed Hp scores. The abscissa represents the heterozygosity rate, and the ordinate represents the distribution of zHp; μ, mean, σ and standard deviation.

**FIGURE 4:**
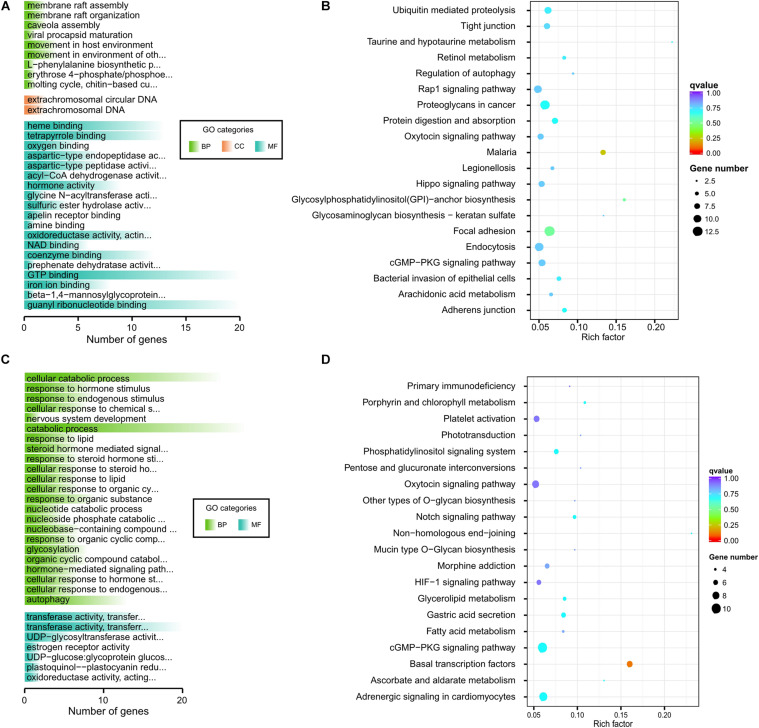
GO and KEGG analysis of horn traits. **(A)** Go categories of horned group. **(B)** KEGG of horned group. **(C)** Go categories of polled group. **(D)** KEGG of polled group.

To better explore the functional areas closely related to horn traits, we grouped 32 samples of high-depth sequencing, dividing eight polled AMS and eight polled AHS into one group (polled group), and eight horned AMS and CMS into another (horned group). We pairwise F_*ST*_ and θπ ratio in a sliding window across the autosomes between polled and horned group to identify the regions of selective sweeps, which using for subsequent GO and KEGG functional enrichment analysis (Figures 3, 4). At the same time, we conducted a horned/polled comparison within one breed, i.e., a horned/polled comparison within the AMS (Supplementary Figure S6). The horned/polled groups were also compared between CMS and AHS (Supplementary Figure S7). By GO analyzing the genes enriched in each pathway, we found that Relaxin receptor 2 (*RXFP2*) was enriched in the neuroactive ligand–receptor interaction pathway, which indicates that this pathway may control the formation of horns. This pathway can be used to study what substances *RXFP2* is controlled by, and which components *RXFP2* controls. We found that *RXFP2* is mediated by G protein, which promotes the activation of adenylate cyclase and the accumulation of cAMP, and the increase of cAMP content will promote cell metabolism, differentiation and the production of certain physiological functions. Several genes related to cAMP were found in this study, such as *FRYL*, *FDX*, *RIC3*, *GDNF-AS1*, and *GRM4*. Preliminary prediction is that *RXFP2* receives the signal to promote the accumulation of cAMP, and cAMP and other factors activate enzymes related to the production of keratin, and then begin to form horns. Additionally, from Figure 3D it can be clearly seen that on chromosome 10, the Z-transformed population differentiation (ZF_*ST*_) is significantly higher than the threshold (ZF_*ST*_ ≥ 0.05), and the Z-transformed pooled heterozygosity (ZHp) of this part of the region is also partially reduced, which indicates that this region is related to the selected traits (horned and polled).

**FIGURE 5:**
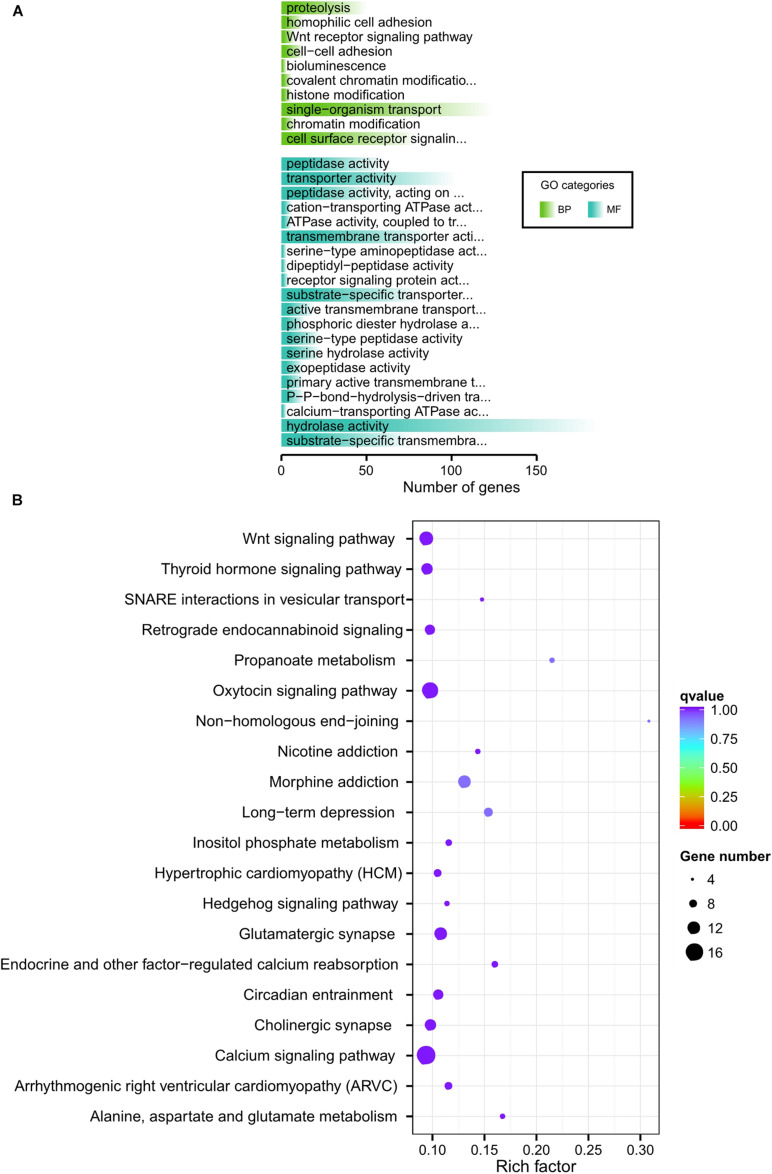
GO and KEGG analysis of adaption.

Fine-wool sheep are subjected to long-term, high-intensity artificial selection as part of the breeding process. To better explore the degree of selection of fine-wool sheep adaptive traits, we explored the correlation between fine-wool sheep horn traits and adaption. We analyzed plateau adaption by dividing the sheep into different groups. Firstly, eight horned AMS samples (high altitude) and eight horned CMS (low altitude) were grouped to analyze environmental adaptability (Supplementary Figure S4). The results showed that the biological process of gene enrichment in the selected region is single-organism transport. Among the significantly enriched pathways, pathways related to thyroid hormone secretion and metabolism were found, and pathways related to pyruvate metabolism, propionate metabolism and glycosaminoglycan degradation were also found. Thyroid hormone secretion and metabolic pathways indicate basal metabolic levels, indicating a significant correlation with highland adaption. Propionic acid metabolism may be related to body fat synthesis, because propionic acid is the main prerequisite for gluconeogenesis in ruminants, and gluconeogenesis generates a large amount of NADPH, providing raw materials for lipid synthesis. Secondly, eight polled AMS (high altitude) and eight polled AHS (low altitude) were grouped to analyze environmental adaption (Supplementary Figure S5). We found that some pathways, such as the AMPK signaling pathway and glutamatergic synapse were related to energy metabolism. Additionally, non-homologous, end-joining signaling pathways related to DNA repair and UV resistance that are significantly associated with high-altitude, low-oxygen adaptive traits were discovered. Finally, we grouped 16 high-altitude merino sheep (high altitude, no distinction between horns and no horns) and 16 low-altitude individuals (CMS and AHS) together to analyze environmental adaptability (Figure 5). The above results all present signal pathways related to adaptability, and explain the gene function related to adaptability.

### Candidate Genes

The goals for breeding fine-wool sheep focus on the traits such as horned/polled and plateau adaptability. Understanding how domestication has shaped patterns of genetic variation could reveal rapid evolution that is triggered by human selection (Andersson et al., 2008; Ciccia et al., 2012; Yuan et al., 2012). We obtained 71 candidate genes embedded in selected regions for closely related to the above traits for subsequent analysis (Supplementary Table S4). Among these, candidate genes were related mainly to mismatch repair (such as *RFC4* and *MSH6*), metabolism (such as *PIGM*, *PIGO*, and *PIGN*), vascular smooth muscle contraction (such as *PLA2G2E* and *PPP1R12A*), and cardiac muscle contraction (such as *ATP1B2* and *RYR2*). Also, *PIGL* and *MET*, which is consistent with previous reports of genes related to adaption (Li et al., 2013). Genes involved in metabolism, mismatch repair, vascular smooth muscle contraction, and others are all indicators of maintaining genomic stability against UV radiation and molecular adaptation under hypoxia. In *RXRG*, *TSHB*, and *HSP90B1* genes, the annotation is related to thyroid hormone signaling, which is also closely related to the immunity of fine-wool sheep to adapt to extreme environmental adaptations. Genes related to thermogenesis, such as *CPT2*, *NDUFV3*, and *NPR1*, can provide an effective guarantee for the characteristics of fine-wool sheep adapting to cold environments.

In order to further reveal the genetic basis of the adaption and horn characteristics of fine-wool sheep, the candidate gene haplotype, genotype frequency, and SNP distribution in different breeds of each candidate gene were analyzed in depth (Supplementary Figures S1–S3). Haplotype-based genetic analyses have been used in human, animal and plant genetics research. Such haplotypes are normally inferred either from a genome sequence, or through linkage or association analysis. In comparison with using individual SNPs, haplotype-based analysis can reduce false discovery rates because it performs fewer association tests (Hamblin and Jannink,2011). The accumulated data showed that seven genes (*PLA2G2E*, *CTBP1*, *THBS1*, *TLR2*, *PPP1R12A*, *MET*, and *CREM*) (*Nei and Li*, 1979; Wang et al., 2015) demonstrated significant haplotype blocks (Supplementary Figure S1). For example, the *TLR2* gene contains two significant haplotype blocks in the segment NC_019474.2: 3841774 to NC_019474.2: 3853952, one of which is located at NC_019474.2: 3841774 - NC_019474.2: 3843106 and the other is located at NC_019474.2: 3853952 - NC_019474.2: 3852343.

At the same time, population genotype frequency statistics of all candidate genes showed that the genotype frequency of *PP1R12A*, *THBS1*, *TLR2*, and *PLA2G2E* was significantly different between the breeds (Supplementary Figure S2). Our results suggest that regions that are affected by natural selection are smaller than those affected by artificial selection in fine-wool sheep. These selected genes also exhibited significant differences in F*_*ST*_* and Tajima’s D. This result quantitatively shows that artificial selection is more powerful than natural selection in shaping the genome, which results in rapid changes in phenotypic and/or behavioral traits. Genes such as *NDUFS4*, *TSC1*, *ITPR1*, *NLK*, *ADCY8*, *RPS6KA2*, *CAMK2D*, and *RPS6KA5* all exhibit highly selected characteristics (Supplementary Figure S3).

Genotypic and allelic frequencies, expected heterozygosity (*He*), effective allele number (*Ne*), polymorphism information content (*PIC*), Hardy–Weinberg equilibrium (*HWE*) testing *p*-value of single-nucleotide polymorphisms (SNPs) were estimated as measures of genetic variability. Expected heterozygosity (He, also called gene diversity) and PIC values are both measures of genetic diversity among genotypes in breeding populations, which sheds the light on the evolutionary pressure on the alleles and the mutation rate a locus might have undergone over a time period. In our study, we selected that six important genes, such as *MSH6*, *PIGO*, *PIGM*, *CPT2*, *CRB3*, and *TLR2 for genotyping analysis* (Supplementary Table S5). Based on the value of average, *PIC*, *PIGO*, *PIGM*, and *CPT2* were categorized as having medium genetic diversity (0.25 < *PIC* < 0.50) for all four breeds. *MSH6* and *CRB3* were categorized as having low genetic diversity (*PIC* < 0.25). It is novelty that three nucleotide varieties of *CRB3* are in the Hardy–Weinberg equilibrium, and one is a homozygous genotype that is unique to alpine merino sheep [such as g.15560554 (CC), g.15561685 (TT), and g.15562465 (TT)]. These statistical results exhibited that these genes might be a potential candidate gene to improve fine-wool perfecter traits, and the SNP could be used as molecular markers in early marker-assisted selection (MAS) in fine-wool breeding program.

## Discussion

Genomic comparisons between closely related species provide insights into the genetic basis of mammalian divergence and adaptation (Kong et al., 2008). In the present study, we performed whole-genome sequencing of 120 fine-wool sheep, including four Chinese indigenous fine-wool sheep breeds from continuous altitudes from the Songliao Plain (low altitude, 800 m) to the Qilian mountains (high altitude, 3200 m). We cataloged millions of SNPs of each breed for evolutionary and genetic research. Specifically, this is the first study to characterize the genetic diversity of fine-wool sheep in China. We analyzed the population using PCA, STRUCTURE, and NJ-tree. Our population structure analyses revealed that the four fine-wool sheep breeds have a relatively separate genetic background. Overall, the partitioning of genetic diversities of the breeds is consistent with their geographic distributions. In STRUCTURE result, it found the existence of introgression among QHS and AMS, but this needs more intensive research to explain. This could be caused by living in Qilian mountainous region of the northwest region and joint breeding.

Most studies of artificial selection in sheep have focused on single-gene analyses arising from phenotype-driven studies. We conducted selective sweep mapping for the breeds from different altitudes and identified several candidate regions with a high extent of differentiation on the genome scale. A few genes that are positively selected may be involved in the adaption-related signaling pathway, as in other mammals, such as humans (Huerta-Sánchez et al., 2014; Lorenzo et al., 2014), Tibetan mastiffs (Li et al., 2014), and horses (Hendrickson, 2013), which are mainly used to examine responses to low-oxygen environments. *ATP1B2*, *RYR2*, *HSP90B1*, and *NPR1* as functional candidate genes related to “response to hypoxia” were reported in Tibetan wild boars (Li et al., 2013). In this study, the genes identified are mainly related to energy metabolism and nucleic acid repair. Adapted to the high altitude-induced extremely harsh conditions, such as hypoxia, low temperature, high solar radiation, and lack of food resources, low-oxygen environments impose severe selective pressure on species living at high altitude. Kijas et al. analyzed 74 sheep breeds worldwide, one of which was a Chinese native breed, Tibetan sheep. The study identified the strongest selected candidate gene, *RXFP2*, in response to breeding for the absence of horns (Kijas et al., 2012). We also identified this gene in chromosome 10 that was enriched in the neuroactive ligand–receptor interaction pathway, which indicates that this pathway may control the formation of horns. Several genes related to cAMP, such as *FRYL*, *FDX*, *RIC3*, *GDNF-AS1*, and *GRM4*, were predicted as important factors for horn forming. In a recent study, selection signatures in merino (and merino-derived) sheep breeds using genome-wide SNP data and two different approaches, a classical FST-outlier method and an approach based on the analysis of local ancestry in admixed populations, were investigated. The most reliable signals were observed on OAR10 (*FRY* and *RXFP2*) (Megdiche et al., 2019). In our study, we also captured *FRYL* and *RXFP2*. Taken together, the above results further contribute to decipher the genetic basis underlying the merino phenotype. In sheep, *FRY* has been suggested as a key candidate gene for the piebald phenotype in merino (Garcia-Gamez et al., 2011) and has been suggested to be associated with the black spot phenotype in valley-type Tibetan sheep (Wei et al., 2015), with differences in coat color pigmentation distribution between the Awassi and Afec-Assaf sheep (Seroussi et al., 2017). Some research suggested *FRY* to be a candidate gene affecting wool quality (Zhang et al., 2013). It may indicate the hidden physiological basis correlation between wool traits and horn type.

Selection signature refers to the traces left by both artificial and natural selection pressures on the genome of a species. Artificial selection has played an important role in the domestication of animals. Approximately 11,000 years ago, domestic animals began to appear in Arab countries in the Middle East. Sheep are one of the earliest domesticated domestic animals (Zeder, 2008), and the earliest sheep breeding was carried out for meat production. From approximately 4000–5000 years ago, domesticated sheep were also bred for secondary products, such as wool (Chessa et al., 2009). Domestication involves remodeling the behavior, morphology, and genetics of animals. It has been reported that by scanning the genomic selection signal of the three sheep populations (AMS, CMS, and AHS), artificial selection changed the sheep’s coat color, horn type, and growth and development traits (Wang et al., 2015). The FST method estimates the variation of the population through DNA polymorphism, is currently based on the Bayesian layered model, and is widely used to identify selection signals (Weir and Cockerham, 1984; Riebler et al., 2008). Using this method, 14 new selection loci for resistance and susceptibility to gastrointestinal nematodes have been found in sheep (McRae et al., 2014), the gene ABHD2 related to emphysema and the gene BMP2 related to the bone morphology and body type of animals (Kijas et al., 2012). Comparing the whole-genome sequence of different breeds of fine-wool sheep in different habitats in China can provide an in-depth insight into the distinct evolutionary scenarios occurring under natural selection for adaptability and occurring under artificial selection for performance traits. This study highlights the value of genome sequencing of phenotypically divergent breeds of the same species for examining genomic patterns of adaptation and horn.

## Conclusion

Our study showed that the genetics of QHS clustered more closely with AMS than other breeds, which indicates that they have a closer genetic relationship and probably share a similar domestication history. Fine-wool sheep have been selected by artificial, high-intensity breeding, which has resulted in the selection of genomic regions involving adaption and horn traits. Our results also provide a reference for future gene editing design and genome-wide selection in sheep.

## Data Availability Statement

The original contributions presented in the study are publicly available. This data can be found in NCBI under accession number PRJNA680869.

## Ethics Statement

The animal study was reviewed and approved by the Laboratory Animal Management and Ethics Committee of Lanzhou Institute of Husbandry and Pharmaceutical, Chinese Academy of Agricultural Sciences.

## Author Contributions

TG and YJY conceived and designed the experiments. JL performed the experiments. ZL and ShZ analyzed the data. CY and HZ contributed the reagents, materials, and analysis tools. SH, CN, and YXY prepared the figures and/or tables. SWZ, YC, XW, and BY approved the final draft of the manuscript submitted for review and publication. All authors contributed to the article and approved the submitted version.

## Conflict of Interest

The authors declare that the research was conducted in the absence of any commercial or financial relationships that could be construed as a potential conflict of interest.

## Supplementary Material

Supplementary Figure 1 Haplotype analysis of candidate genes.

Supplementary Figure 2 Genotype frequency of candidate genes in different breeds.

Supplementary Figure 3 Genes embedded in artificial selected regions in fine-wool sheep related to horn traits.

Supplementary Figure 4 Eight horned alpine merino sheep samples (high altitude) and 8 horned Chinese merino sheep (low altitude) form a group as a horn group to analyze adaption.

Supplementary Figure 5 Eight polled alpine merino sheep samples (high altitude) and 8 polled Chinese merino sheep (low altitude) form a group as a horn group to analyze adaption.

Supplementary Figure 6 Intra-species comparison of horn and polled alpine merino sheep.

Supplementary Figure 7 Inter-species comparison of Chinese merino sheep (horn) and Aohan fine-wool sheep (polled).

Supplementary Table 1 Introduction of the four sheep breeds examined in the present study.

Supplementary Table 2 Number of reads and bases in quality control.

Supplementary Table 3 Read mapping statistics and coverage of depth.

Supplementary Table 4 Candidate genes list.

Supplementary Table 5 Genotypic and allelic frequencies, expected heterozygosity (*He*), effective allele number (*Ne*), polymorphism information content (*PIC*), and Hardy–Weinberg equilibrium (*HWE*) testing *p-*value of single-nucleotide polymorphisms (SNPs) identified in *MSH6, PIGO, PIGM, CPT2, CRB3*, and *TLR2*.
