# Supplementary figures and images for: Selective Sweeps Uncovering the Genetic Basis of Horn and Adaptability Traits on Fine-Wool Sheep in China
Source: Front Genet. 2021 Feb 23;12:604235. doi: 10.3389/fgene.2021.604235 (PMC7940688; doi:10.3389/fgene.2021.604235)

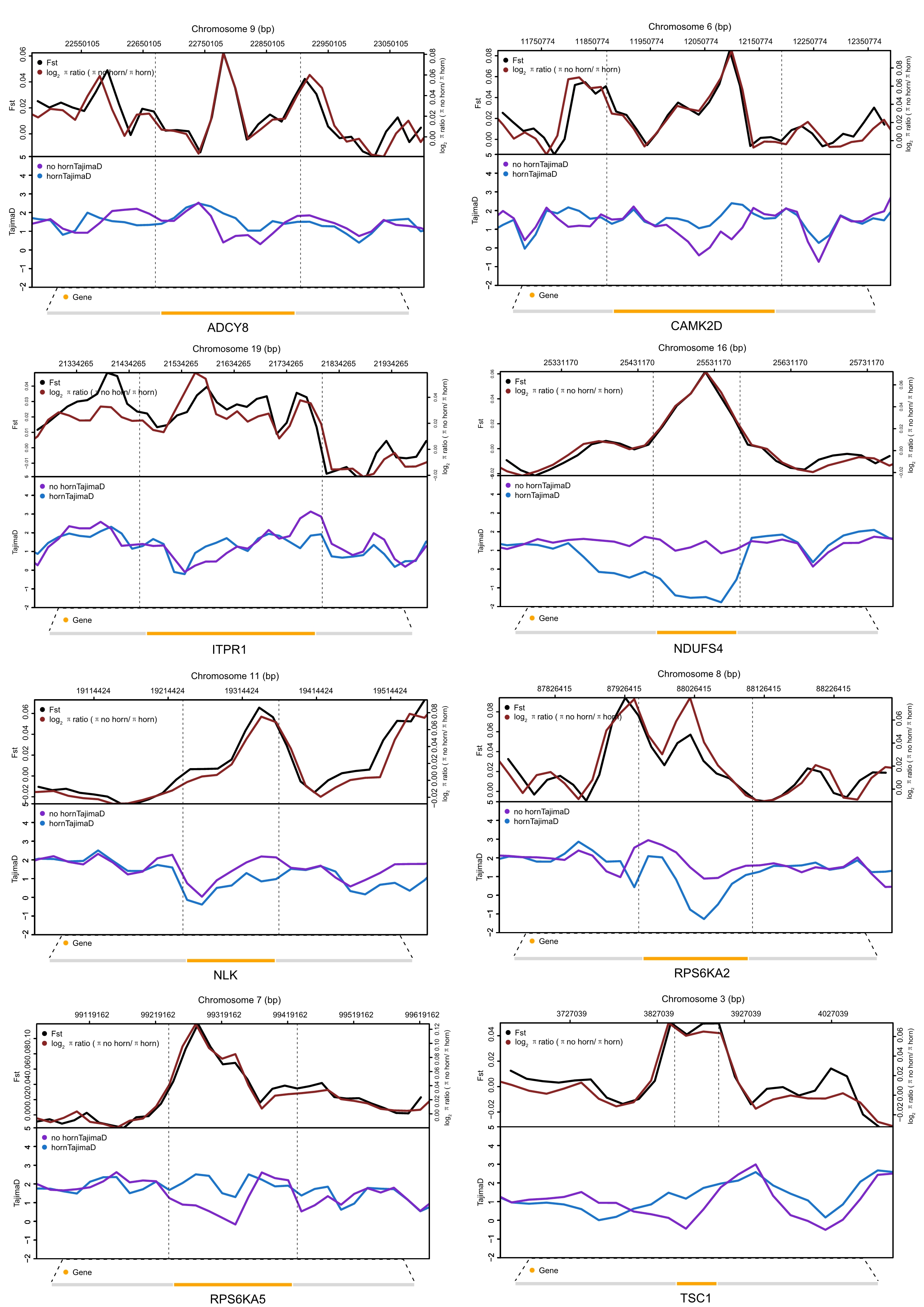

Supplement: Supplementary Figure 1 — Haplotype analysis of candidate genes. [file Image_1.JPEG]

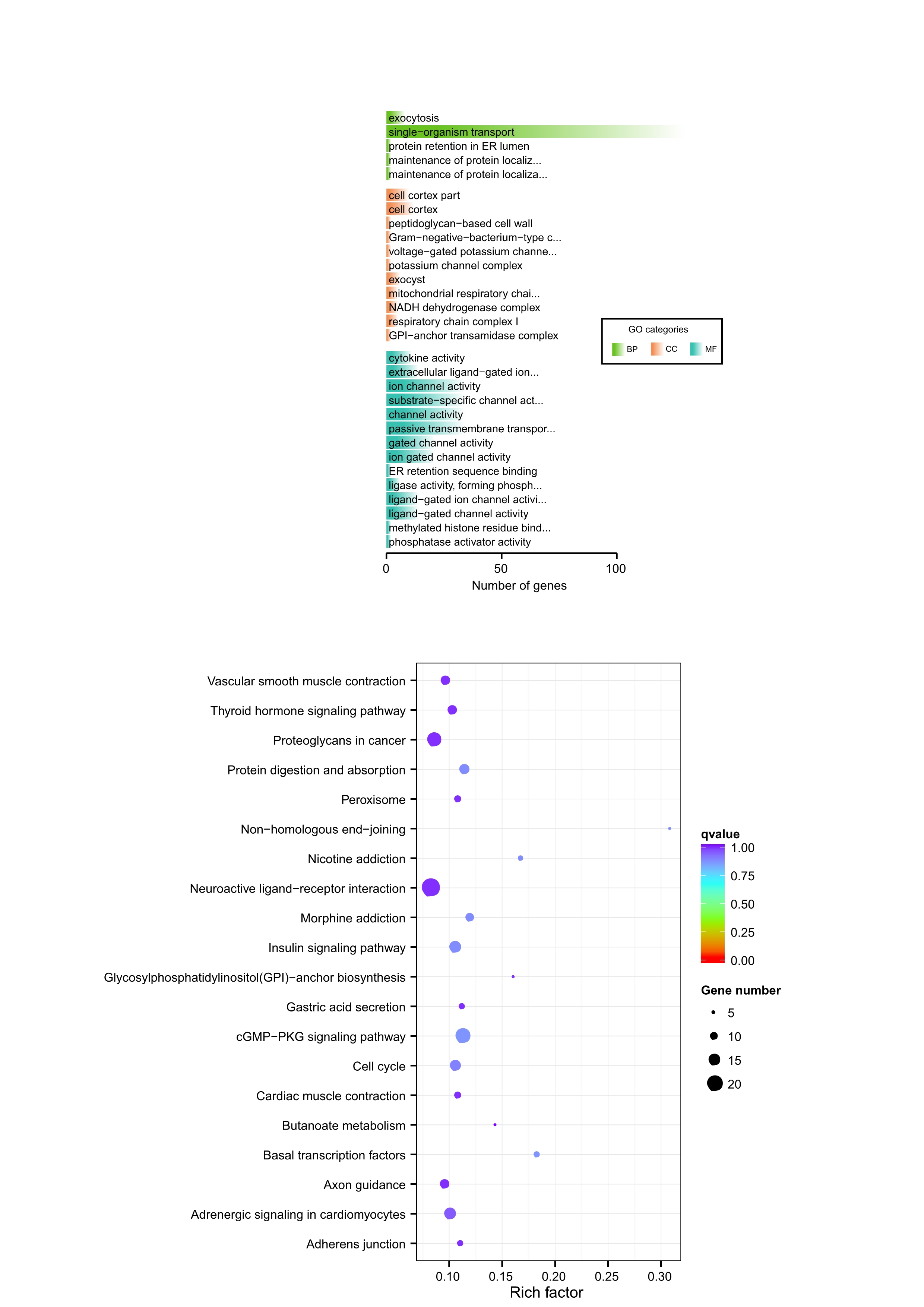

Supplement: Supplementary Figure 2 — Genotype frequency of candidate genes in different breeds. [file Image_2.JPEG]

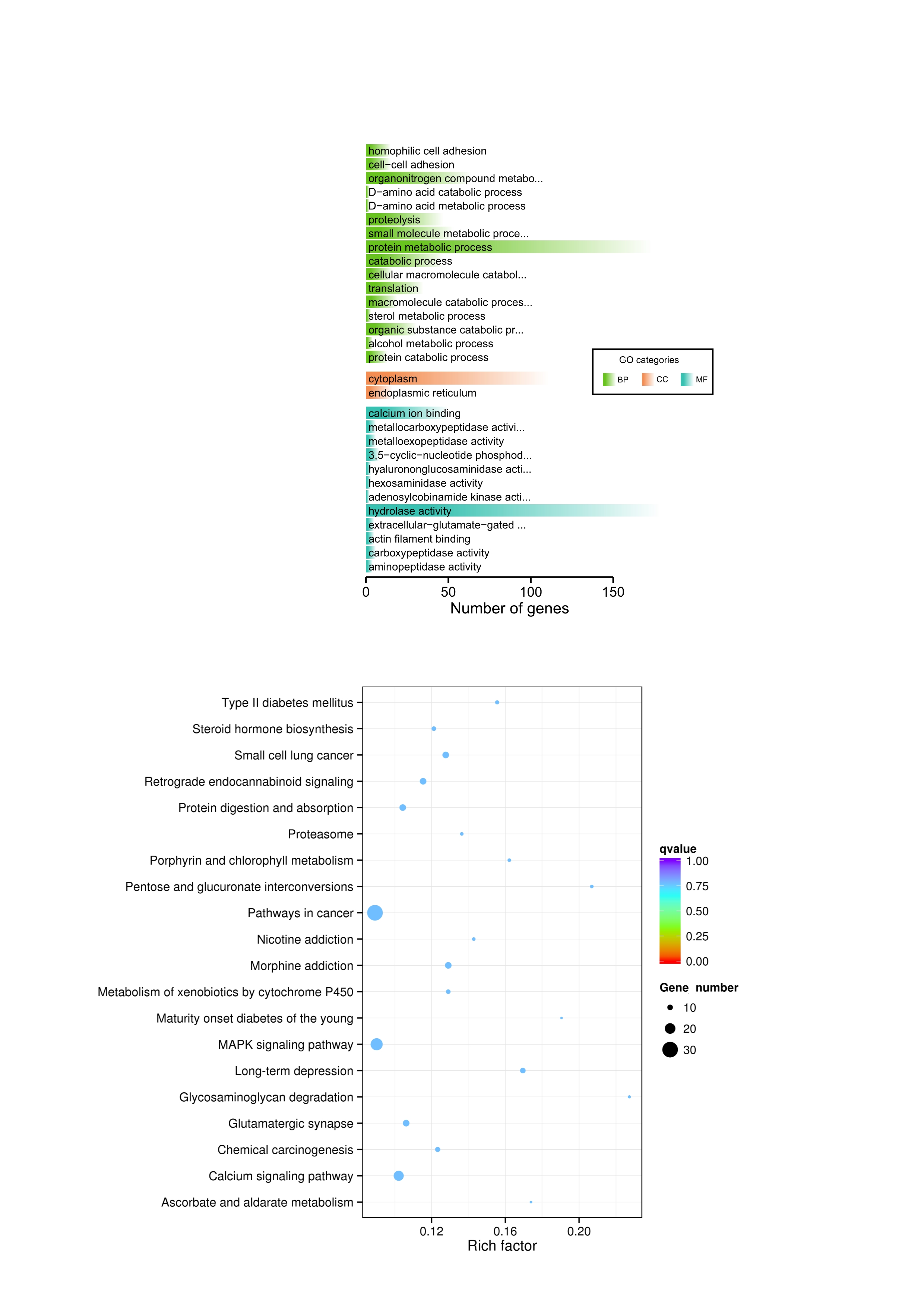

Supplement: Supplementary Figure 3 — Genes embedded in artificial selected regions in fine-wool sheep related to horn traits. [file Image_3.JPEG]

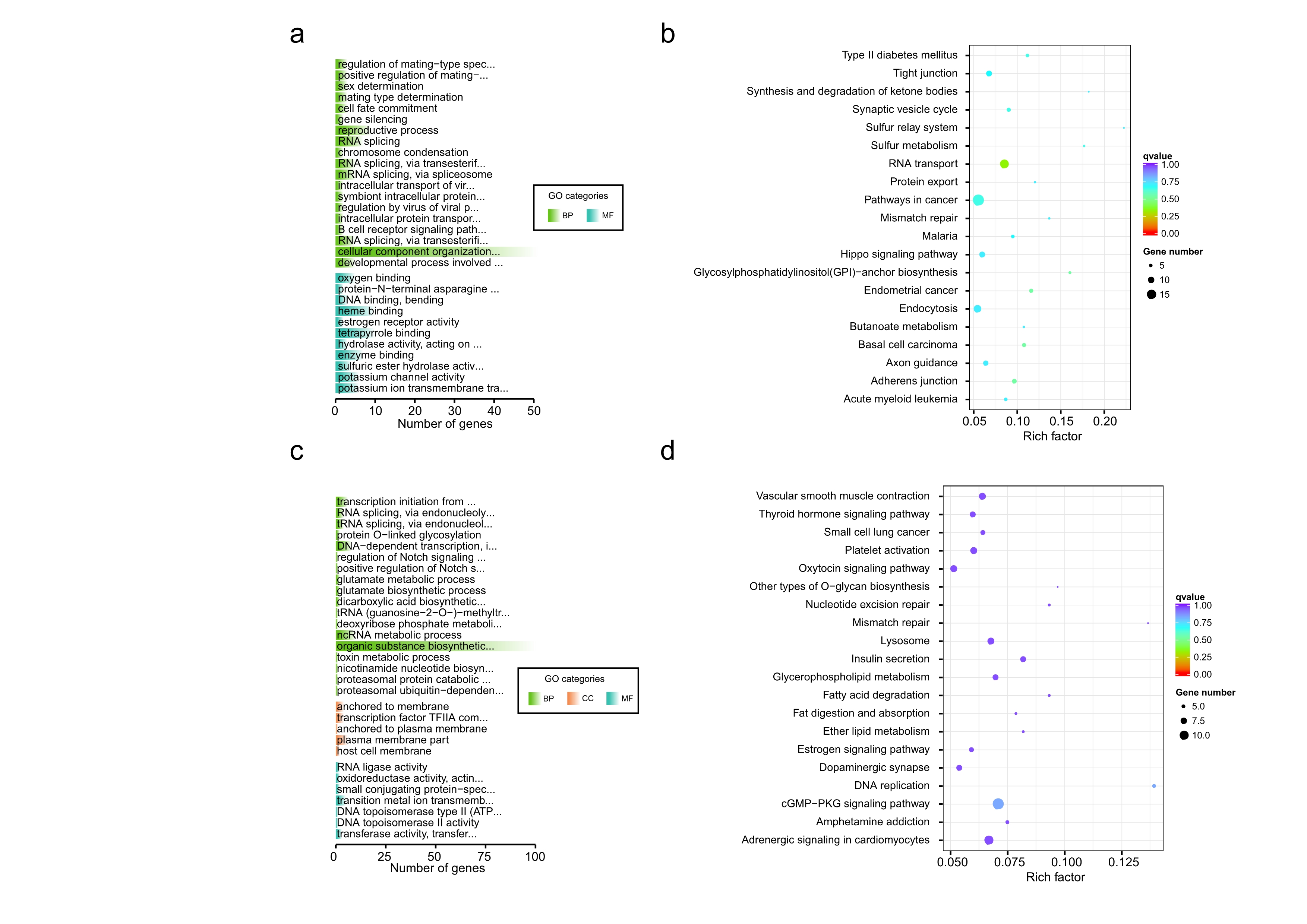

Supplement: Supplementary Figure 4 — Eight horned alpine merino sheep samples (high altitude) and 8 horned Chinese merino sheep (low altitude) form a group as a horn group to analyze adaption. [file Image_4.JPEG]

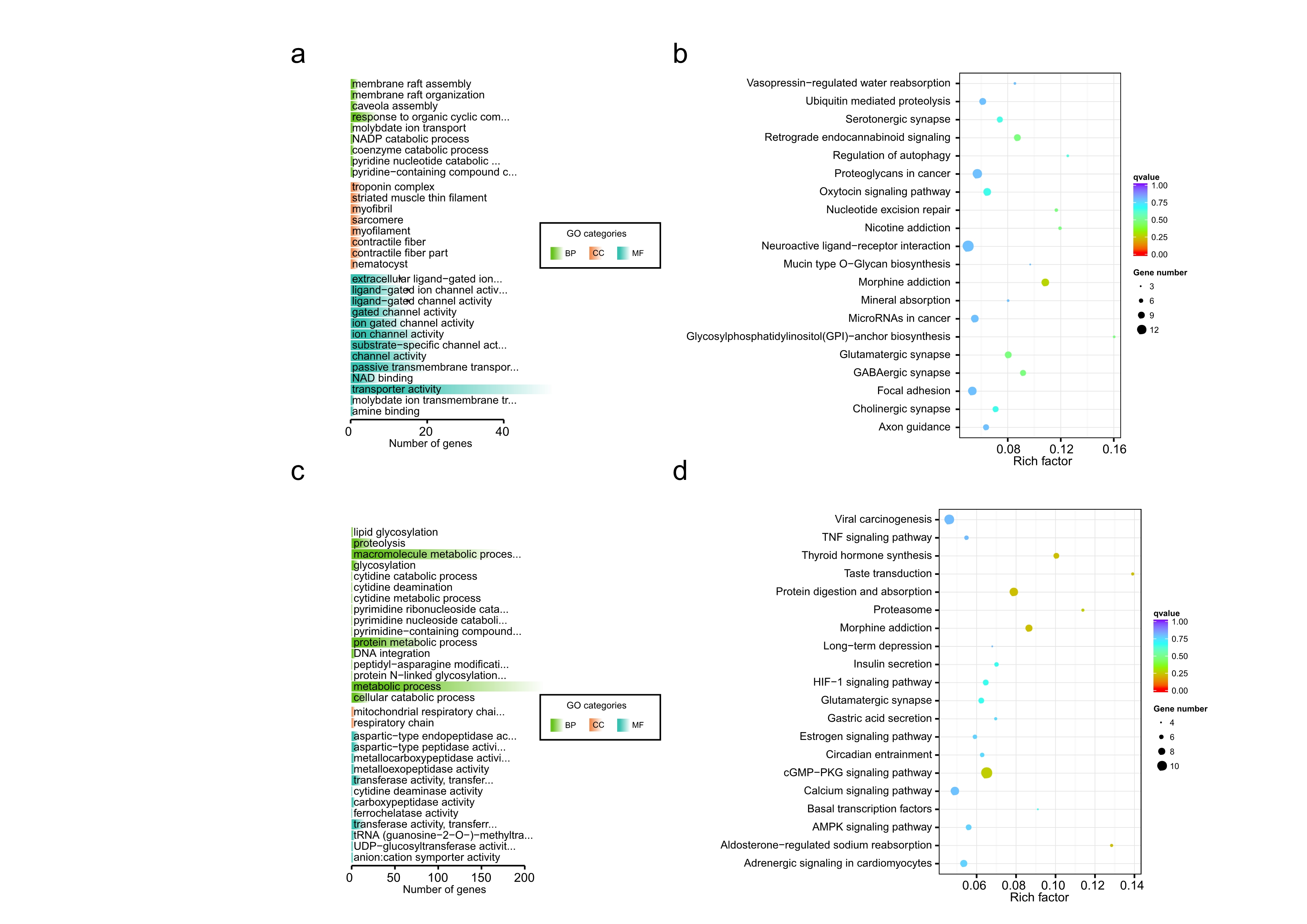

Supplement: Supplementary Figure 5 — Eight polled alpine merino sheep samples (high altitude) and 8 polled Chinese merino sheep (low altitude) form a group as a horn group to analyze adaption. [file Image_5.JPEG]

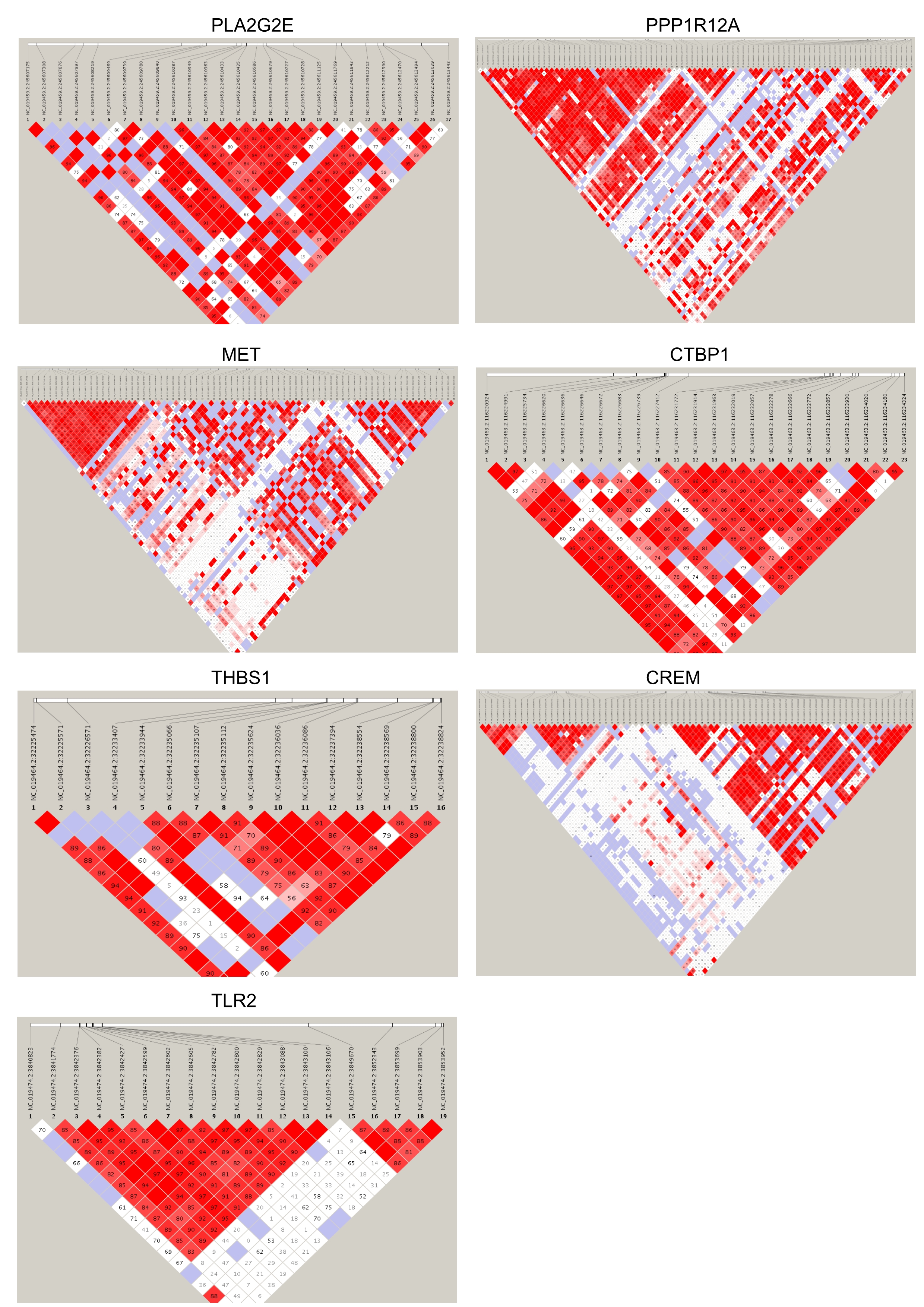

Supplement: Supplementary Figure 6 — Intra-species comparison of horn and polled alpine merino sheep. [file Image_6.JPEG]

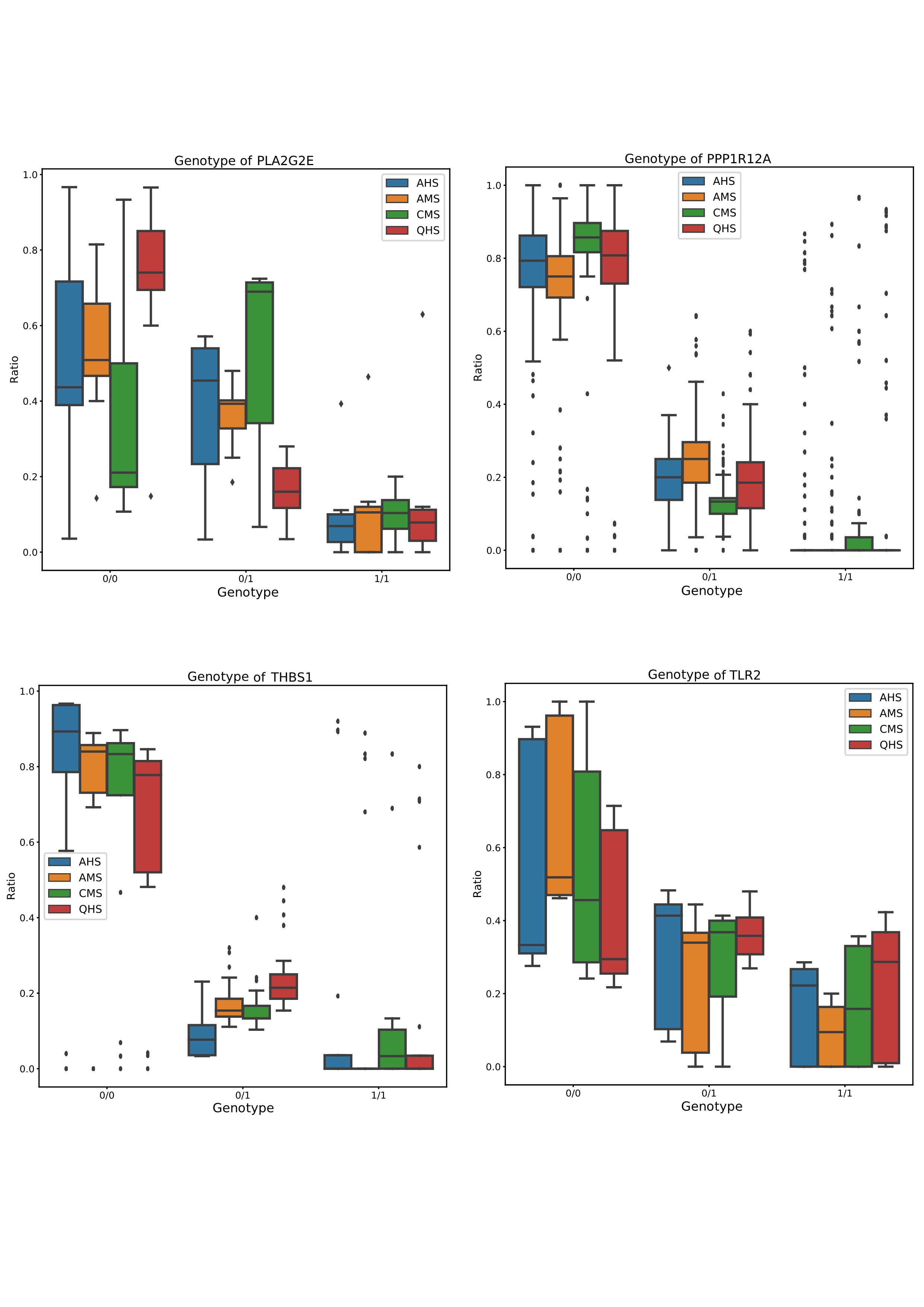

Supplement: Supplementary Figure 7 — Inter-species comparison of Chinese merino sheep (horn) and Aohan fine-wool sheep (polled). [file Image_7.JPEG]
